# An Ensemble Convolutional Neural Networks for Bearing Fault Diagnosis Using Multi-Sensor Data

**DOI:** 10.3390/s19235300

**Published:** 2019-12-02

**Authors:** Yang Liu, Xunshi Yan, Chen-an Zhang, Wen Liu

**Affiliations:** 1 State Key Laboratory of High Temperature Gas Dynamics, Institute of Mechanics, Chinese Academy of Sciences, Beijing 100190, China; liuyang2@imech.ac.cn (Y.L.); lw@imech.ac.cn (W.L.); 2 School of Engineering Science, University of Chinese Academy of Sciences, Beijing 100049, China; 3 Institute of Nuclear and New Energy Technology, Tsinghua University, Beijing 100084, China; 4 The Key Laboratory of Advanced Reactor Engineering and Safety, Ministry of Education, Beijing 100084, China; 5 Collaborative Innovation Center of Advanced Nuclear Energy Technology, Beijing 100084, China

**Keywords:** rotating machinery, fault diagnosis, multi-sensor fusion, convolutional neural network, ensemble model

## Abstract

Multi-sensor data fusion is a feasible technique to achieve accurate and robust results in fault diagnosis of rotating machinery under complex conditions. However, the problem of information losses is always ignored during the fusion process. To solve above problem, an ensemble convolutional neural network model is proposed for bearing fault diagnosis. The framework of the proposed model contains three convolutional neural network branches: one multi-channel fusion convolutional neural network branch and two 1-D convolutional neural network branches. The former branch extracts the coupling features based on multi-sensor data and the latter two branches extract the inherent features based on single-sensor data, which can collect comprehensive fault information and reduce information losses. Furthermore, the support vector machine ensemble strategy is employed to fuse the results of multiple branches, which can improve the generalization and robustness of the proposed model. The experiments show that the proposed can obtain more effective and robust results than other methods.

## 1. Introduction

Rotating machinery is widely used in modern industry. Due to long-time running under complicated conditions such as high speed, heavy load and strong impact, rotating machinery will inevitably have some faults, which can result in enormous losses and serious casualties [1]. Therefore, the fault diagnosis of rotating machinery is necessary to ensure the safe and efficient operation of machinery [2,3].

Traditional fault diagnosis methods are mainly based on model analysis or signal processing techniques. The model-based diagnosis methods emphasize the deep understanding of the dynamic characteristics of rotating machinery. Immovilli et al. [4] conduct a theoretical analysis based on the vibration and current signals. Kerschen et al. [5] provide extensive reviews on model-based analysis of vibrating systems. These methods usually require the design of the explicit mathematical model to simulate the behavior of the machine, while the development of the mathematical model is almost impossible when dealing with modern machines with very complex structures. The methods based on signal processing techniques often utilize signal models, such as power spectrum [6], high order spectrum [6,7,8], composite spectrum [9,10,11], to directly extract the fault features from the measured signals for the classification. Among the various measured signals, vibration signals [12,13] are most popular due to the inclusion of more fault information. In addition to the vibration signals, magnetic flux signals [14] are also used to fault diagnosis. Besides, some researchers have also applied these methods for machine condition monitoring [15,16,17]. However, these methods still rely on the analysis of the mechanical structure and extracting effective fault features is blind and difficult.

Different from traditional diagnosis methods, Intelligent fault diagnosis aims to effectively analyze massive data and automatically provide diagnosis results, which has become a new trend in the field of equipment condition monitoring [18]. Generally, traditional intelligent fault diagnosis of rotating machinery can be regarded as a pattern recognition problem. It can be divided into two steps: feature extraction and pattern classification [19], which can result in two inherent shortcomings—(1) The feature extraction process is difficult in that it relies on advanced signal processing technology and extensive engineering experience [20]. Moreover, the extracted features often do not fully reflect the fault characteristics which limits the application. (2) Current pattern recognition methods in the fault classification, such as artificial neural network (ANN) [21,22] and support vector machine (SVM) [23], belong to the shallow learning model. Such model has no more than one nonlinear transformation [24]. Due to the complex non-linear characteristics of fault signals, the shallow learning model is difficult to effectively learn representative features for fault diagnosis [25,26]. Consequently, it is necessary to build deep-architecture learning model to achieve more efficient and automatic fault diagnosis.

In recent years, deep learning provides a new research hotspot for analyzing and processing the big data, which has made great achievements in the fields of image, finance, meteorology, and natural language [27]. As a typical model of deep learning, a convolutional neural network (CNN) [28] can automatically learn more representative characteristics for fault diagnosis, thus overcoming the inherent shortcomings of traditional intelligent diagnosis methods. CNN has been widely applied in the field of fault diagnosis. However, in most studies [29,30,31,32], the input is only limited to the single-sensor data, which greatly limits the further performance improvement of the CNN fault diagnosis model. To overcome these drawbacks, a feasible method is using the information provided by multi-sensor data.

According to the literature [33], multi-sensor data fusion of fault diagnosis can be divided into three levels: data-level fusion, feature-level fusion and decision-level fusion. Data-level fusion is the lowest level, including Kalman filtering [34], principal component analysis (PCA) [35] and independent component analysis (ICA) [36], and so forth. This fusion methods directly takes data from different sensors as new data sources, which ignores the inner relationship of different sensors [37]. For the feature-level fusion, features from different sensors are extracted and selected by signal processing techniques. The more abundant data from multiple sensors makes the selection of sensitive features more difficult [38]. Decision-level fusion fuses the results of different classifiers to achieve a better decision. Common fusion algorithms include the majority voting [39], SVM [40,41], dempster-shafer (DS) evidence theory [42], random forest (RF) [43] and Bayesian estimation [44]. However, these studies for different levels of fusion generally ignore the coupling relationship between multi-sensor data, which causes information losses [37].

Recently, the CNN models based on multi-sensor data have been used for fault diagnosis. Generally, multi-sensor signals are connected into long signals [38] or arranged into 2-D images [45] as the input of CNN. Furthermore, Gong et al. [46] propose an improved CNN-SVM model by usage of the 2-D signals from multi-sensor data. Chen et al. [47] compare different fusion methods at the input layer of CNN by taking horizontal and vertical vibration signals. These models can effectively extract the coupling features between different sensors, thus achieving better performance than traditional methods based on multi-sensor data. However, on the one hand, they ignore the inherent information of the single-sensor data and fail to effectively fuse the inherent information with the coupling information acquired by the multi-sensor data, resulting in information losses. On the other hand, the generalization of the model is important due to the large inter-class divergence and small divergence between classes of the collected signals. But current research mainly focuses on the optimization of individual network, which limits the generalization of the model. In addition, the splicing or arranging of massive time-domain data may lead to the problem of over-fitting.

To solve the above problems, a novel model named ensemble convolutional neural networks (ECNN) using multi-sensor data is proposed for bearing fault diagnosis in this manuscript. The proposed model, ECNN, can automatically and effectively extract features for classification that gets rid of the dependence on signal processing techniques and diagnosis experience. Besides, a multi-channel fusion convolutional neural network (MCF-CNN) and two 1-D CNNs are designed to construct ensemble model. The coupling features between multi-sensor data are extracted by the MCF-CNN model and the inherent features of the single-sensor data are extracted by 1-D CNN model, overcoming the problem of information losses. Furthermore, to improve the generalization and robustness of the proposed model, the SVM combination strategy is employed to integrate the result of multiple CNN models. The proposed model uses frequency spectrum as the input of CNN, verified based on two typical rolling bearing fault databases.

In Section 2, a brief theory of CNN is introduced. Section 3 describes the main framework of ECNN in detail. In Section 4, the experimental results are analyzed and discussed based on the CWRU database and the Paderborn University database. Finally, the conclusions and outlook are given in Section 5.

## 2. The Standard Convolutional Neural Network

**Figure 1:**
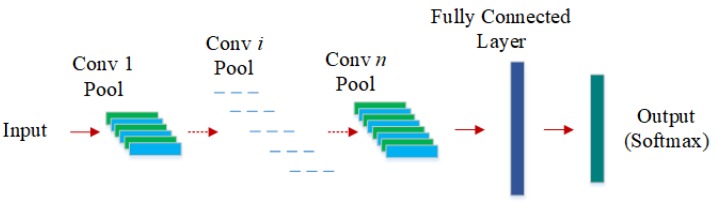
The standard convolutional neural network.

CNN is one of the most prevalent deep learning models in recent years. As shown in Figure 1, CNN processes the input samples through multiple convolutional layers and pooling layers to obtain a series of deep feature maps. Suppose the input of *l*th convolutional layer is x, which belongs to $R^{A\times B}$ and *A* and *B* are the dimensions of the input data. Then the output of the convolutional layer can be calculated as follows:

(1) $$u_{j}^{l}=f\left(k_{j}^{l}*x^{l-1}+b_{j}^{l}\right)$$

(2) f=max(0,x)

where $u_{j}^{l}$ is the linear activation, $k_{j}^{l}$ is a set of kernels of *l*th convolutional layer, * represents the convolutional operation, $x^{l-1}$ represents the feature maps in the previous layer l−1, $b_{j}^{l}$ is the bias vector and *f* is the Relu activation function. The output $u_{j}^{l}$ is conducted by the max pooling operation:

(3) $$u_{j}^{l}=up\left(u_{j}^{l}\right)$$

where $u_{j}^{l}$ is the output feature maps of pooling layer and up(·) represents the up-sampling function of max pooling.

After several operations of convolution and pooling, deep feature maps are expanded into fully connected layer:

(4) $$x^{l}=f\left(k^{l}x^{l-1}+b^{l}\right)$$

where $x^{l}$ is the fully connected layer, $k^{l}$ is the weight, $x^{l-1}$ represents the feature vector in the previous layer l−1, $b^{l}$ is the bias vector.

For the output $x=\{x_{1},\;x_{2},\;\ldots ,\;x_{I}\}$ of last fully connected layer, softmax function achieves the mapping between the fully connected layer and the target output:

(5) $$y_{i}=\frac{e^{x_{i}}}{\sum e^{x}}$$

where $y_{i}$ is the predicted probability belonging to the *i*th class, $x_{i}$ is the *i*th output neuron of last fully connected layer.

## 3. The Proposed Model

**Figure 2:**
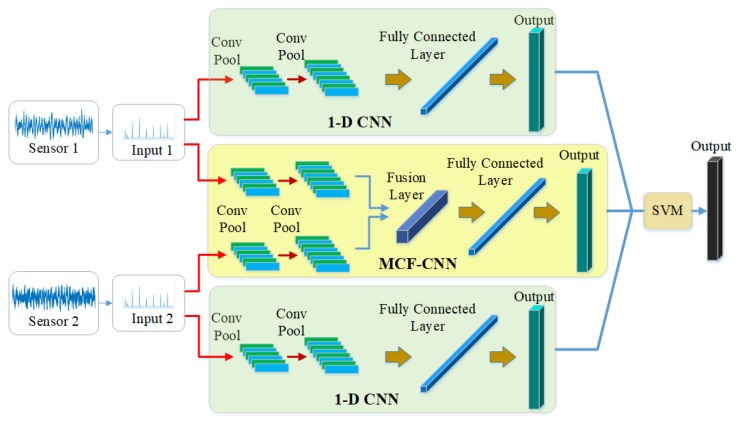
The framework of the proposed model.

In this manuscript, a novel model, ECNN, is developed for bearing fault diagnosis. The input of the model is the frequency spectrum of the collected signals so that we select the one-dimensional convolutional neural network (1-D CNN) with the one-dimensional filter as the kernel of CNN. It is clearly seen that the framework contains three CNN branches based on two-sensor data. Figure 2 shows the overall framework of the proposed model. The MCF-CNN branch is used to extract the coupling features between the two-sensor data and the two 1-D CNN branches focus on the contribution of the single-sensor data. Thus, more comprehensive fault information can be collected for fault diagnosis, overcoming the problem of information losses. The SVM combination strategy is employed to give the final results by fusing three CNN branches. More details of the proposed model are described in the following subsection.

### 3.1. Multi-Channels Fusion Convolutional Neural Network

**Figure 3:**
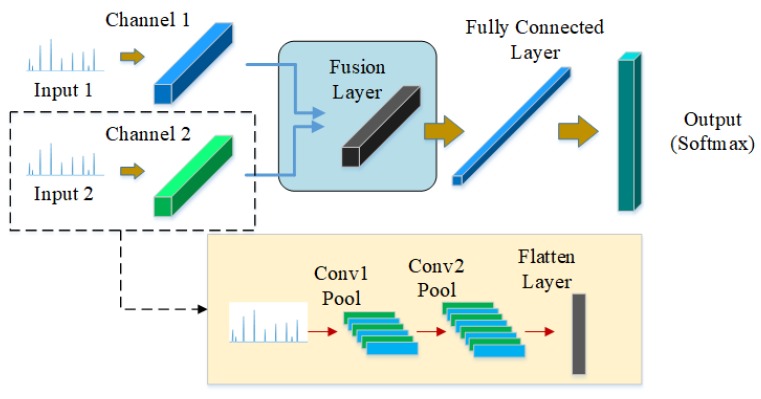
Multi-channel convolutional fusion neural network.

As shown in Figure 3, MCF-CNN has two independent channels at the input layer which process multi-channel data separately. After multiple convolution and pooling operations, the deep features from multi-sensor data are fused at the fully connected layer. The main idea is that the two independent channels based on different sensors can extract the coupling features. Then the two channels are fused to enhance the fault information that facilitates classification. Finally, the classification is accomplished at the end of the network. It can be assumed that the MCF-CNN has better performance than the CNN with single-channel input because the multi-channel inputs are simultaneously trained under the same learning framework and the parameters of different channels can be jointly optimized during the training process.

**Figure 4:**
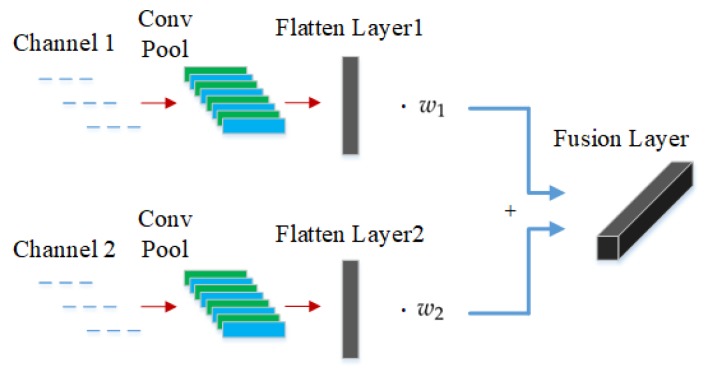
Fusion layer to combine two feature vectors.

Figure 4 shows the fusion layer of MCF-CNN. Each input channel of MCF-CNN processes the input data through the convolution and pooling operations, calculating a number of feature maps. The two channels are set at the same network structure. The number of convolutional layers, the size of the kernel and the activation function are identical. At the end of each channel, the extracted feature maps are expanded into a one-dimensional feature vector, which is flatten layer. Next, the fusion layer combines the 1-D feature vector of the two channels. Denote the two feature vectors as $v_{1}$ and $v_{2}$. The dimensions of $v_{1}$ and $v_{2}$ should be equal, then the fused feature vector z can be obtained

(6) $$z=w_{1}\cdot v_{1}+w_{2}\cdot v_{2}$$

where $w_{1}$ and $w_{2}$ are the fusion weight of the feature vectors $v_{1}$ and $v_{2}$, respectively, which are learned during the training process. The dimension of the fusion weight is consistent with the dimension of the feature vector. The relationship can be determined by the two weights, which is beneficial to extract the coupling features.

After the fusion layer, two fully connected layers are used to classify the input signals. The first fully connected layer has hundreds of neurons and the number of neurons in the last fully connected layer corresponds to the categories of classification tasks. The softmax function is used to convert the vector of the last fully connected layer into a probability distribution form.

### 3.2. The Construction of Ensemble Convlutional Neural Networks

The traditional fault diagnosis methods based on multi-sensor data ignore the coupling information between signals. The CNN fault diagnosis models based on multi-sensor data focuses on the coupling information between the signals and ignores the inherent information of the single-channel sensor. These methods all result in information losses. In addition, the performance of CNN fault diagnosis model is often limited due to single network framework. Ensemble learning is a new technique, which uses multiple individual learners and a certain combination strategy to get better results than each individual learner. Recently, a lot of ensemble learning methods have been applied for machinery fault diagnosis. Thus, ensemble learning of ECNN is constructed for fault diagnosis.

To collect more comprehensive fault information, the 1-D CNN model and the MCF-CNN model are used to construct ECNN. Since the databases contain the data from two sensors, two 1-D CNN models and one MCF-CNN model can be obtained. The input of the 1-D CNN is single sensor data, while the input of the MCF-CNN is the data from two sensors which are accepted by the two channel of MCF-CNN respectively.

### 3.3. The Fusion Strategy

The next step is to design a fusion strategy to combine the results of three CNN branches. Among the common fusion strategies, the majority voting is a widely used fusion method for ease of execution. However, the majority voting treats each classifier equally, resulting in poor performance when there are fewer classifiers. Obviously, it is not appropriate to use this method here because there are only three classifiers. The SVM is a popular machine learning method for classification, regression because of the small structural risk. In addition, the SVM model with kernel function can also learn the non-learning relationship of the input data. Thus, The SVM model with RBF kernel is chosen as the ensemble learning algorithm to fuse the results of three CNN branches.

For the *n*th samples, define the predicted probability that belong to the *i*th class of the *m*th CNN as $y_{in}^{\left(m\right)}$ and m∈{1,2,3}, which can also be regarded as deep feature representations of the input sample. The deep feature representations of $y_{in}^{\left(1\right)}$, $y_{in}^{\left(2\right)}$ and $y_{in}^{\left(3\right)}$ are employed as the input of the SVM ensemble learning algorithm. The input can be expressed as follow:

(7) $$y_{n}=\left\{y_{1n}^{\left(1\right)},\;\ldots ,\;y_{In}^{\left(1\right)},\;\ldots ,\;y_{in}^{\left(j\right)},\;\ldots ,\;y_{1n}^{\left(J\right)},\;\ldots y_{In}^{\left(J\right)}\right\}$$

where n∈N, *N* represents the number of the train samples. On the training set, we use the deep feature representations $y_{n-train}$ of the *n*th training sample as the input and the real label ${\hat{y}}_{n-train}$ of the samples as the output to learn the SVM ensemble learning algorithm. On the testing set, the deep feature representations $y_{n-test}$ are used as the input of the SVM ensemble learning algorithm and then the prediction ${\tilde{y}}_{n-test}$ of the samples can be obtained. The accuracy of the classifier is can be expressed:

(8) $$accuracy=\frac{1}{N}\sum_{n=1}^{N}I({\tilde{y}}_{n-test}={\hat{y}}_{n-test})$$

where ${\hat{y}}_{n-test}$ represents the real label of the *n* sample on the testing set and I(·) represents the indicator function.

### 3.4. The General Procedure of the Proposed Model

**Figure 5:**
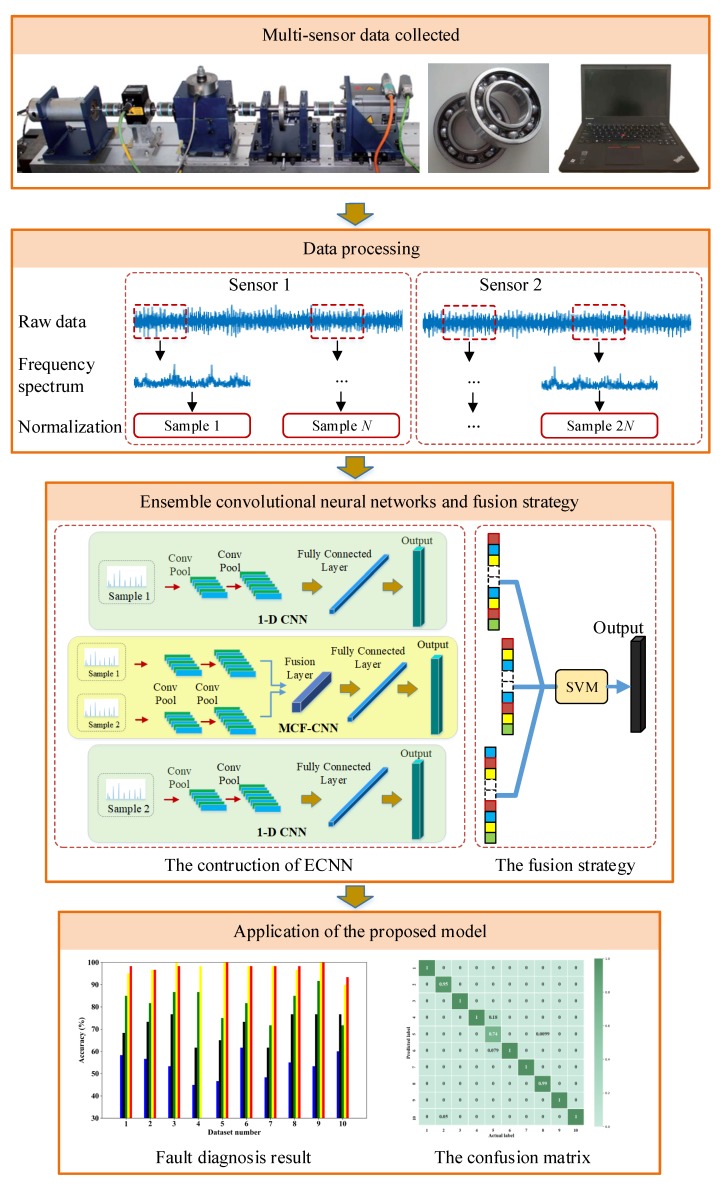
The flowchart illustrating of the proposed model (the test rig in the first frame is from Paderborn University [48]).

This manuscript develops a new model called ECNN for bearing fault diagnosis. Figure 5 gives the flowchart illustrating of the proposed model and the general procedure can be summarized:

Step 1: The signal acquisition devices collect data from multiple sensors.

Step 2: The collected data are divided into training and testing set and the raw signals are divided into a series of segments. The frequency spectrum of the segment is used as the input of the CNN model.

Step 3: The MCF-CNN based on multi-sensor data and two 1-D CNNs based on single-sensor data are designed to construct ECNN based on training set.

Step 4: The SVM ensemble algorithm is employed to combine the results of three CNN branches.

Step 5: Validate the performance of the ECNN based on the testing set.

### 3.5. Discussion of the Proposed Model

Based on the typical model of deep learning, convolutional neural network, the proposed model fuses the multi-sensors data as two level. The advantage of the proposed model is can be summarized as follows:

1. The proposed model does not require complex mathematical models and does not rely on signal processing techniques and expert experience compared with the traditional fault diagnosis methods.
2. Compared with intelligent diagnosis methods, the proposed model does not require the process of complex feature extraction and feature selection. Besides, the deep learning framework can effectively extract more useful fault information for classification, which can further improve the diagnostic accuracy. Similarly, the new network structure, MCF-CNN, has the better ability to extract features.
3. The MCF-CNN model fuses multi-sensor data at the feature level and ECNN fuses the results of three CNN branches at decision level, effectively overcoming the problem of information losses during the fusion process.

## 4. Experiments

To demonstrate the effectiveness, the proposed model is tested based on two typical rolling bearing databases. The CNN models are created based on the framework of TensorFlow 1.4. The Nesterov Adam algorithm [49] is used to optimize the CNN models and the learning rate of the optimizer is 0.0005. All the experiments are carried out on a computer with Intel CPU E5-2680 and an NVIDIA Tesla T4 GPU.

### 4.1. Data Processing

The input of current CNN fault diagnosis models is usually a short segment of raw signals and the CNN models based on multi-sensor data fusion often directly combine segments from multiple sensors to long sample or arrange the segments to a 2-D image as the input of CNN models. Obviously, the length of segment has an impact on the performance of the CNN models. If the length of segment is too long, the splicing or arranging of much time domain data can result in over-fitting and the waste of resources and time. If the length is too short, the model appears to be under-fitting, which cannot learn effective fault features.

**Figure 6:**
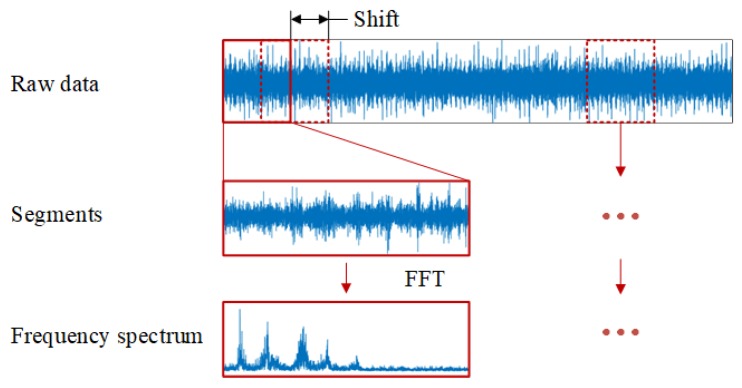
The steps of data preprocessing.

According to the literature [50], in the collected signals of the rotating machine, information about the fault characteristics often resides in the low frequency components and useless information generally exists in the high frequency components. Thus, traditional fault diagnosis methods usually convert time domain signals to the frequency domain through Fourier Transform (FT) and only the frequency components under 1 kHz are used for fault diagnosis. Figure 6 gives the three steps of data preprocessing. First, the raw signals are divided into several segments with a shift size. Second, each segment is transformed into the frequency domain by Fast Fourier transform (FFT). Finally, the frequency components under 1 kHz are kept and normalized as the input of CNN models.

The min-max normalization strategy is used to map each sample into [0, 1] interval. The equation is as follows:

(9) $$\hat{x}=\frac{x-min(X)}{max(X)-min(X)}$$

where x represents the sample, $\hat{x}$ represents the normalized result of x, max(·) and min(·) represent the maximum and minimum function, respectively, and X represents the training set.

### 4.2. Case 1: Experiment on CWRU Database

#### 4.2.1. Dataset Description

**Figure 7:**
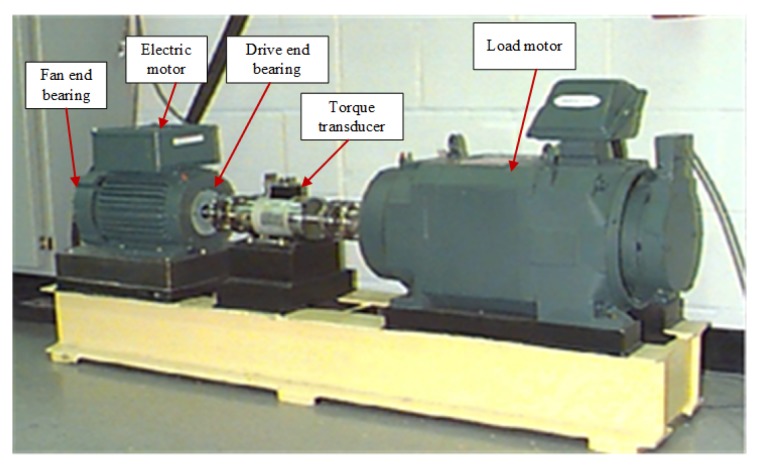
The test rig in the Case Western Reserve University lab [51].

In this subsection, the bearing data for experiment comes from the Bearing Data Center of Case Western Reserve University [51]. The test rig is shown in Figure 7, which mainly consists of an electric motor (Reliance Electric 2HP IQPreAlert motor), a torque transducer and a load motor. Each test bearing is installed in the test motor and tested under four different loads (0, 1, 2 and 3 hp, 1 hp = 0.7355 kw). Two accelerometers are installed at the drive end (DE) and the fan end (FE) of the motor casing to collect vibration signals at a sampling frequency of 12 kHz. Single point fault is introduced to test bearings using electro-discharge machining with a diameter of 0.007, 0.014, 0.021 and 0.028 inches (1 inch. = 25.4 mm). More details of the database were described in Reference [52].

As shown in Table 1, ten kinds of fault bearings are collected for fault classification, including different fault types, fault severities and fault orientations. Based on the four different loads, for each fault, bearing data of three loads are randomly selected as training data and the left for testing and ten different datasets are collected. Bearing data of each load has approximately 12,000 points and each segment has 1200 points. Therefore, each dataset contains 3000 training segments and 1000 testing segments, whose low frequency components are used as the training and testing input.

#### 4.2.2. Experiment and Analysis

The excellent performance of the proposed model is proved in comparison with traditional fault diagnosis methods, including SVM, RF and AdaBoost algorithm. The input of traditional methods is the connected frequency spectrum of the two-sensor signals. In addition, a multi-sensor data fusion method based deep convolutional neural network (DCNN) [38] is also chosen for comparison and the raw vibration data from the two sensors is connected as the input of the DCNN model. Based on the deep CNN model, this method can learn features from raw data and optimize a combination of different fusion levels adaptively to satisfy the requirements of any fault diagnosis task.

**Figure 8:**
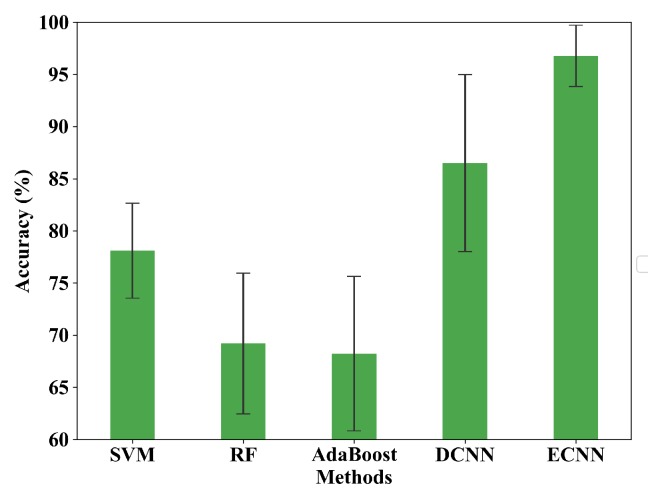
Testing average accuracy of SVM, RF, Adaboost, DCNN and ECNN in case 1.

Ten trials are carried out based on the datasets mentioned in the previous subsection. Figure 8 shows the results of comparison and Table 2 gives the testing average accuracy in detail. It can be seen that traditional fault diagnosis methods are not effective compared with the CNN models. The average accuracy of RF, AdaBoost and SVM is only 69.2%, 68.23% and 77.25%, respectively, which is far from the needs of the industrial application. Comparatively, the average accuracy of DCNN is 86.50%, exhibiting an evident advantage over the traditional methods. However, the drawback is that the standard deviation is large (8.47%), which shows that the generalization of the individual network is limited. Among all the five methods, ECNN has the highest average accuracy (96.78%) and the smallest standard deviation (2.93%).

**Figure 9:**
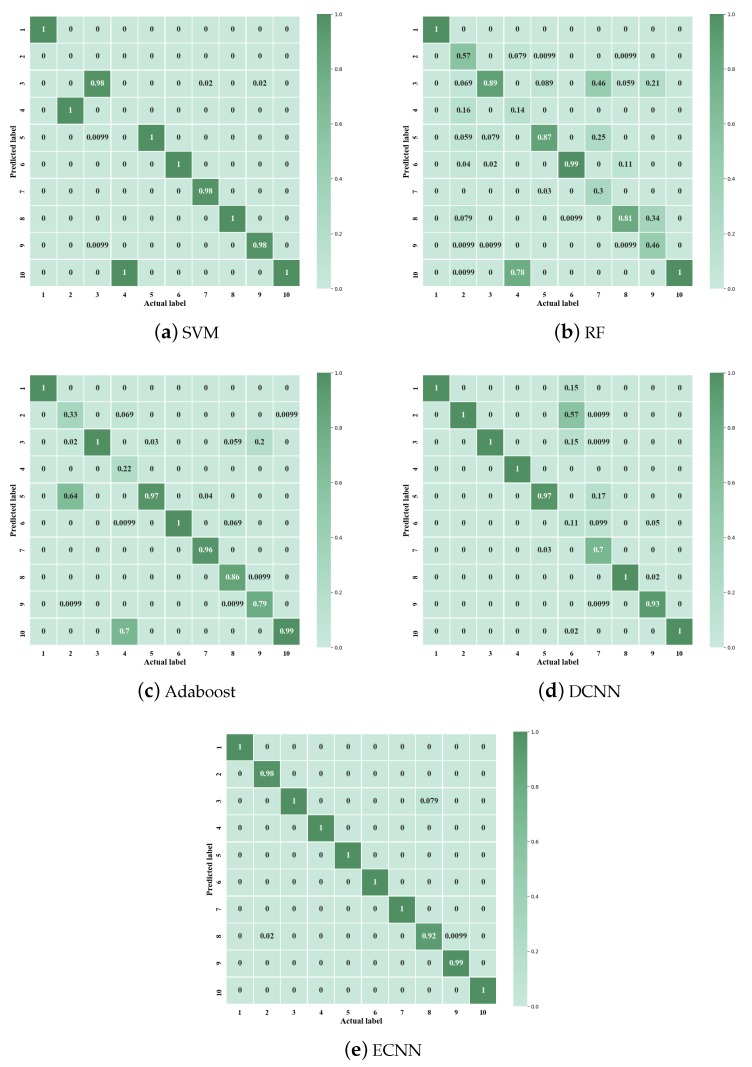
Confusion matrix of different methods in case 1.

Figure 9 gives the confusion matrix of different methods for the first trial. The ordinate axis of the confusion matrix represents the actual label of each class and the horizontal axis represents the predicted label. It can be seen from Figure 9a–c that SVM cannot distinguish the class of 2 and 4. The class 2 is the ball fault and class 4 is the out race fault. In addition to the class 2 and 4, RF and Adaboost cannot accurately identify other classes, which explains why the accuracy of the two methods is lower than SVM. From Figure 9d,e, DCNN shows the low testing accuracy only in class 6 and 7, both of two classes has the same fault types and fault orientations. However, ECNN can accurately distinguish each class.

Through the above experiments, two conclusions can be made. On the one hand, the proposed model has higher accuracy than traditional methods. The reason is that the CNN models can automatically learn more effective features from the input data, while traditional methods rely heavily on manual feature extraction. For traditional methods, the selection of sensitive features is generally time-consuming, blind and subjective so that diagnosis results are generally poor. On the other hand, compared with the direct decision of DCNN, three CNN branches of ECNN based on different sensors and different networks can make joint decisions, which not only ensures the high diagnostic accuracy but also has good generalization.

### 4.3. Case 2: Experiment on Paderborn Database

#### 4.3.1. Dataset Description

**Figure 10:**
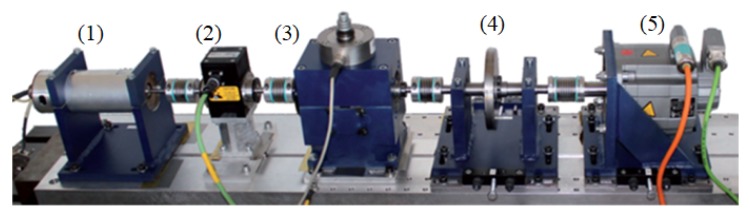
The test rig of the Paderborn database [48].

The experimental data is from the Chair of Design and Drive Technology at Paderborn University [48]. As shown in Figure 10, the test rig includes: (1) an electric motor (Hanning-Motor SD4CDu8S-009/425W/Y230V), (2) a torque measurement module, (3) a rolling bearing test module, (4) a flywheel and (5) a load motor (Siemens-Motor 1FT7062-1AF70-1DG1). The test bench is a modular system and the current signals are collected at a sample rate of 64 kHz. Three health conditions of ball bearings are provided: healthy, inner race fault and outer race fault. Both inner and outer race fault in bearings contain two groups of damages: artificial and real damages. Bearings with real damages are obtained by an accelerated lifetime test. The more details of the damages can be obtained in Reference [53].

The test rig can be operated under different operating conditions. Bearings are run at a speed of 1500 r/min with a load torque of 0.1 N·m and a radial force on the bearing of 1000 N. In order to get closer to industrial applications, the dataset in Table 3 of healthy bearings with real damages were collected, including a total of 15 bearings. For each bearing, the current signals of two channels are measured and each channel collects 20 samples. Therefore, there are a total of 100 samples for each health condition and 80 samples of each are randomly selected as training data and the remaining 20 samples for testing. Ten combinations are chosen to test the model.

The sample is the raw current data of 256,000 points, which is sliced into 60 subsamples. Each subsample has 5120 points with a shift size of 1024. The frequency spectrum of these subsamples is the input of the CNN models. During the testing, the subsamples from the same raw data are voted for the final result. The detailed situation of healthy, inner race fault and outer race fault bearings is shown in Table 4, Table 5 and Table 6. Therefore, a training set of 14,400 subsamples and a test set of 3600 subsamples are obtained.

#### 4.3.2. Experiment and Analysis

**Figure 11:**
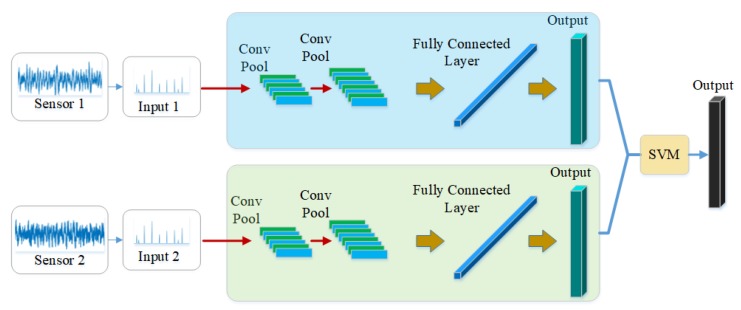
The fusion convolutional neural network.

The experiments are divided into two parts. The first part is the comparison among the proposed model and the traditional methods mentioned in Section 4.2.2. In the second part, the comparison among ECNN, three CNN branches and a fusion convolutional neural network (FCNN) is carried out. As shown in Figure 11, FCNN is chosen here because it has a similar structure and the same input as MCF-CNN. Through the comparison, we can explain the reasons for the excellent performance of ECNN.

**Figure 12:**
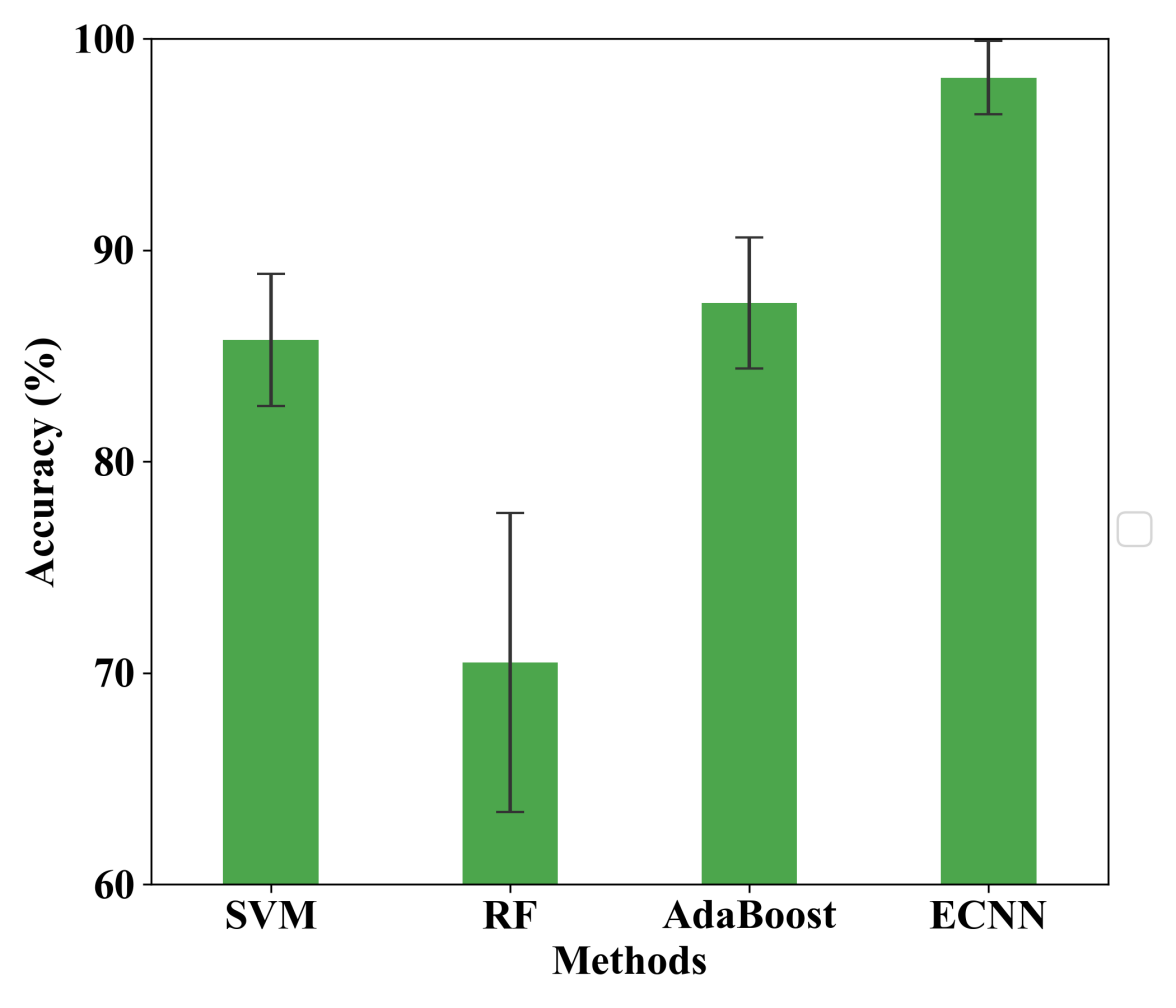
Average accuracy of SVM, RF, AdaBoost and ECNN in case 2.

In the first part, ten trials are carried out. Figure 12 shows the comparison of SVM, RF, AdaBoost and ECNN. The testing accuracy is given in Table 7. Consistent with the results based on the CWRU database, with the highest accuracy (98.17%) and the smallest standard deviation (1.74%), ECNN shows apparently better performance than the other four methods, which demonstrates the good robustness of ECNN. However, difference also exists between the results of the two databases that SVM (85.75%) and AdaBoost (87.50%) show higher accuracy than RF (70.5%).

**Figure 13:**
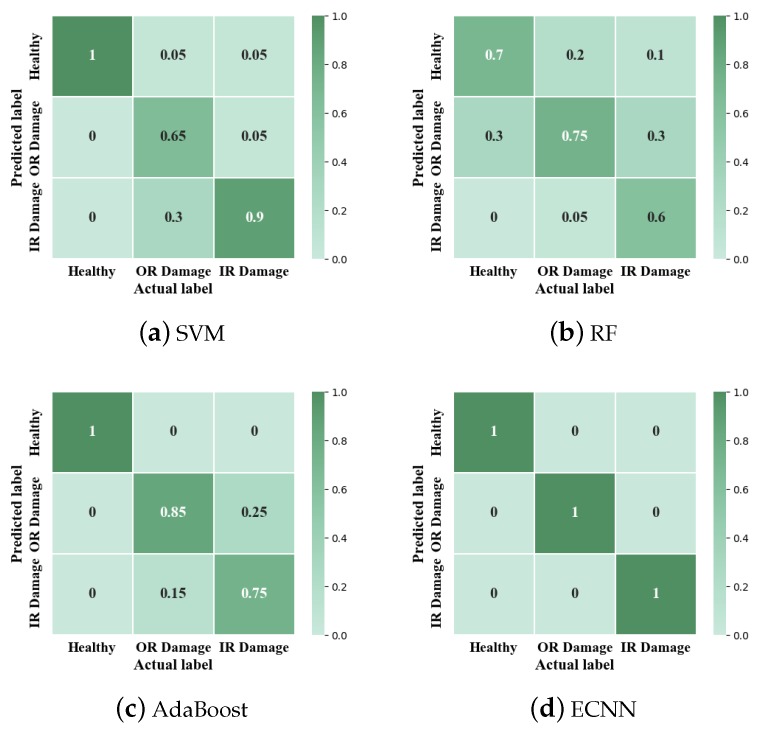
Confusion matrix of different methods in case 2, OR = Outer race; IR = inner race.

As shown in Figure 9, the confusion matrix of different methods is given. From Figure 13a–c, it can be found that the judgment of the healthy condition and inner race damage is the main reason for the different performance of the three methods. In addition, AdaBoost has higher accuracy in outer race damage than the other two methods. From Figure 13d, ECNN has high accuracy of 100% in each class.

**Figure 14:**
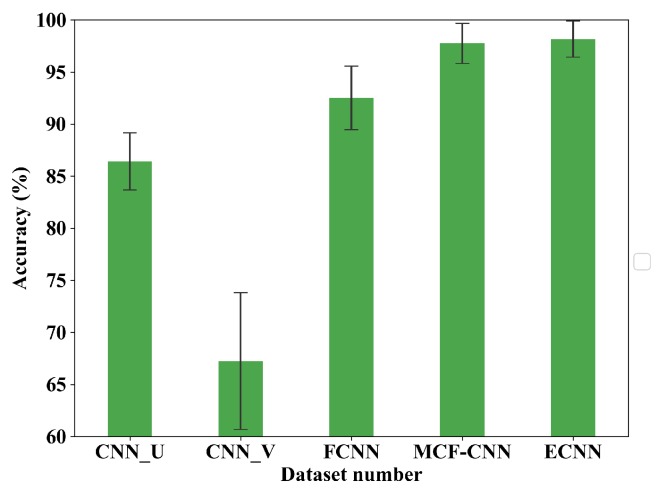
Average accuracy of CNN_U, CNN_V, FCNN, MCF-CNN, ECNN.

**Figure 15:**
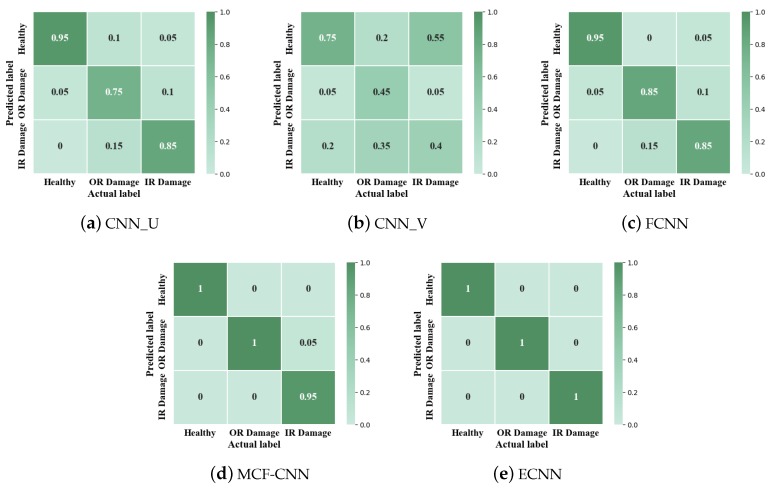
Confusion matrix of CNN_U, CNN_V, FCNN, MCF-CNN, ECNN.

In the second part, two current signals are denoted as U and V and define the 1-D CNN based on U and V as CNN_U and CNN_V, respectively. Table 8 and Figure 14 show the results of the five models. The accuracy of CNN_U is 86.42% and CNN_V is 67.25%, while the accuracy of MCF-CNN is 97.75%. Obviously, the performance of MCF-CNN is much better than the two 1-D CNN models, illustrating the advantages of the model based on multi-sensor data fusion. Besides, MCF-CNN also has higher accuracy than FCNN (92.5%), which shows the effectiveness of the new structure of MCF-CNN. Among all the five methods, ECNN has the highest average accuracy and the small standard deviation. From Figure 15, it can be seen that the low accuracy happens in healthy class and inner race class of CNN_V.

The reasons for the above results can be explained as follows. FCNN can be regarded as ECNN without the branch of MCF-CNN, while the performance of ECNN is better than FCNN. Besides, the accuracy of MCF-CNN is sightly lower than ECNN. Thus, it can be inferred that the excellent performance of ECNN is mainly attributed to MCF-CNN and the CNN_U and CNN_V branches slightly improve the accuracy of ECNN.

#### 4.3.3. Visualization

Recently, the CNN models have developed a variety of complex structures, which make the interpretation of the internal mechanism of the CNN model very difficult. However, with the visualization in the invisible layers, some interesting phenomena can help us understand the mechanism [54].

**Figure 16:**
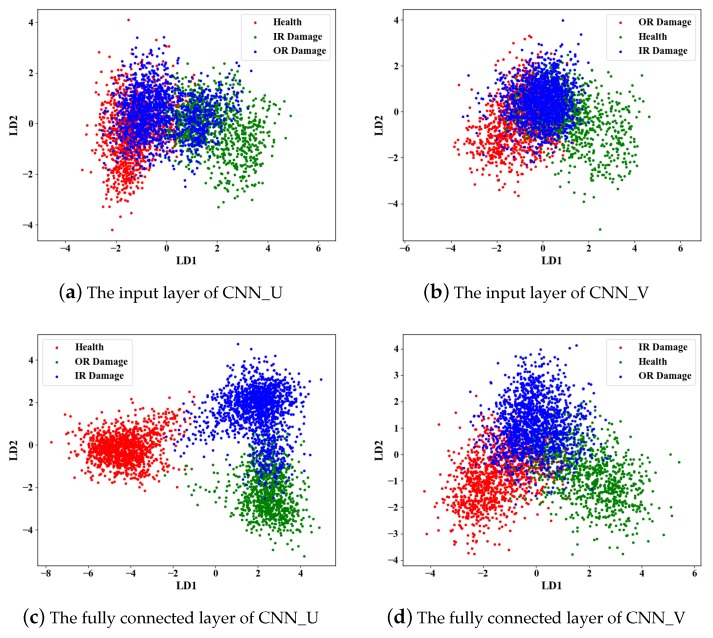

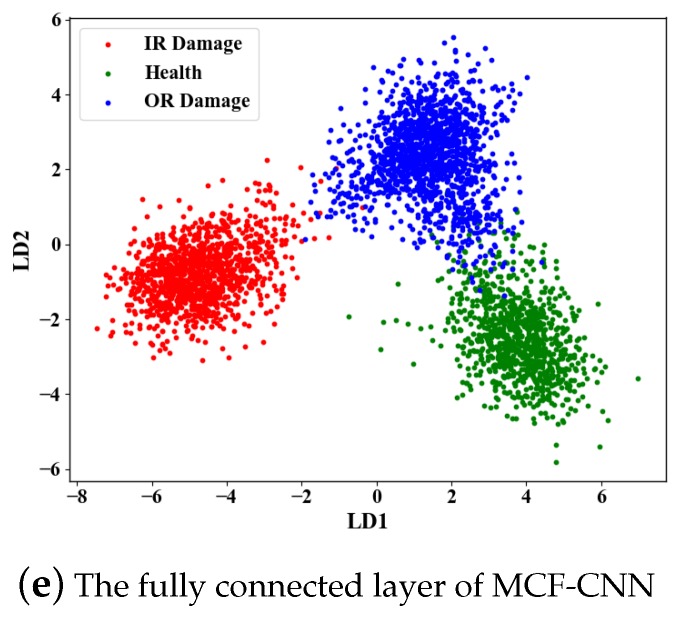
2D-LDA projection of the learned features.

In this manuscript, linear discriminant analysis (LDA) is used to extract two features for visualization. As shown in Figure 16, the visualization of different layers and different networks is given and LD1 and LD2 represent the first two principle components obtained by LDA. Some interesting phenomena can be easily found. First, Figure 16a,b are the visualizations of the input layer of CNN_U and CNN_V. It can be seen that the conditions can not be distinguished at all. Therefore, extracting and selecting sensitive features is difficult, which is the main reason for the poor performance of traditional methods. Second, Figure 16c is the visualization of CNN_U in the first fully connected layers. The healthy condition is easily distinguished from the other two damage conditions, while the two damage conditions can not be distinguished from each other clearly. The visualization of CNN_V in the first fully connected layers is shown in Figure 16d. There are no clear boundaries among the three conditions in that the accuracy of CNN_V is lower than the other branches. Third, Figure 16e is the visualization of MCF-CNN in the first fully connected layers. Obviously, the three conditions can be easily distinguished, which means that MCF-CNN can extract more effective fault features compared to the other two CNNs.

### 4.4. Discussions

1. The experiments are carried out based on two typical rolling bearing fault databases: CWRU database and Paderborn University database. The collected data of the former is vibration signals and the latter is current signals. For the two groups of experiments, the proposed model has much higher accuracy and smaller standard deviation compared with the traditional methods, which proves that the proposed model is effective and has better generalization. What is more, the results of DCNN on the two databases are quite different, while the results of ECNN are extremely consistent, demonstrating the good robustness of the proposed model.
2. The second part of the experiment in case 2 illustrates that the excellent performance of the proposed model is mainly from the contribution of the MCF-CNN branch and the left two branches play slightly auxiliary roles. According to the visualization analysis, MCF-CNN can extract more effective features for fault diagnosis.
3. In the literature [50], the deep inception net with atrous convolution (ACDIN) is proposed to diagnose real bearing faults by only relying on artificial bearing data sets, which achieves high diagnostic accuracy of 96% on Paderborn University database. Different from ECNN, the input of ACDIN is raw vibration signals from single sensor. In the literature [43], a deep random forest fusion (DRFF) technique by using acoustic emission signals and vibration signals is proposed to address fault diagnosis of gearboxes. Similar to FCNN, two deep Boltzmann machines extract the sensitive features of the two signals separately and RF fuses the deep features for classification. However, DRFF ignores the coupling information between the two signals. In comparison, MCF-CNN can be regarded as a feature-level fusion model to extract the coupling features and ECNN is constructed by three CNN branches that can be considered as a decision-level fusion model to collect information comprehensively. This ensures the excellent performance of the proposed model.

## 5. Conclusions

In this manuscript, a novel model ECNN using multi-sensor data is developed for bearing fault diagnosis that get rid of the dependence on signal processing techniques and diagnosis experience. The MCF-CNN model fuses multi-sensor data at the feature level and ECNN fuses the results of three CNN branches at decision level, effectively overcoming the problem of information losses during the fusion process. The proposed model is applied on two typical databases, and the results show that the proposed model has higher accuracy and better generalization than traditional intelligent methods.

The CNN models using multi-sensor data in the field of bearing fault diagnosis are still in the exploratory stage and there are still many problems to be solved, such as the fusion of “heterogeneous” sensors. In the future, we will explore a more efficient fusion method so that these methods can be applied in the modern industry as soon as possible.

## Figures and Tables

**Table 1:** Description of the bearing working condition in the case 1.

Condition	Fault Diameter (Inches)	Fault Orientation	Loads	Traing\Test Samples Number	Label
Normal	0	-	0, 1, 2, 3	300\100	1
Ball	0.007	-	0, 1, 2, 3	300\100	2
Inner race	0.007	-	0, 1, 2, 3	300\100	3
Outer race	0.007	Centered @6:00	0, 1, 2, 3	300\100	4
Outer race	0.007	Orthogonal @3:00	0, 1, 2, 3	300\100	5
Outer race	0.007	Opposite @12:00	0, 1, 2, 3	300\100	6
Outer race	0.014	Centered @6:00	0, 1, 2, 3	300\100	7
Outer race	0.021	Centered @6:00	0, 1, 2, 3	300\100	8
Outer race	0.021	Orthogonal @3:00	0, 1, 2, 3	300\100	9
Outer race	0.021	Opposite @12:00	0, 1, 2, 3	300\100	10

**Table 2:** Diagnosis results of different methods in case 1.

Methods	Average Accuracy (%)	Standard Deviation (%)
SVM	77.25	8.02
RF	69.20	6.76
AdaBoost	68.23	7.40
DCNN	86.50	8.47
ECNN	96.78	2.93

**Table 3:** Data sets for healthy bearings and bearings with real damages.

Healthy	OR Damgae	IR Damgae
K001	KA04	KI04
K002	KA15	KI14
K003	KA16	KI16
K004	KA22	KI18
K005	KA30	KI21

**Table 4:** Situation of healthy bearings.

Bearing Code	K001	K002	K003	K004	K005
Run-in period	>50	19	1	5	10
Radial load [N]	1000∼3000	3000	3000	3000	3000
Speed [rpm]	1500∼2000	2900	3000	3000	3000
Samples	20	20	20	20	20
Subsamples	1200	1200	1200	1200	1200

**Table 5:** Situation of outer race fault.

Bearing Code	KA04	KA15	KA16	KA22	KA30
Type of damage	Real	Real	Real	Real	Real
Extent of damage	1	1	2	1	1
Damage method	Pitting	Plastic deform	Pitting	Pitting	Plastic deform
Samples	20	20	20	20	20
Subsamples	1200	1200	1200	1200	1200

**Table 6:** Situation of inner race fault.

Bearing Code	KI04	KI14	KI16	KI18	KI21
Type of damage	Real	Real	Real	Real	Real
Extent of damage	1	1	3	3	1
Damage method	Pitting	Pitting	Pitting	Pitting	Pitting
Samples	20	20	20	20	20
Subsamples	1200	1200	1200	1200	1200

**Table 7:** Diagnosis results of different methods in case 2.

Methods	Average Accuracy (%)	Standard Deviation (%)
SVM	85.75	3.13
RF	70.50	7.07
AdaBoost	87.50	3.10
ECNN	98.17	1.74

**Table 8:** Diagnosis results of CNN_U, CNN_V, FCNN, MCF-CNN, ECNN.

Methods	Average Accuracy (%)	Standard Deviation (%)
CNN_U	86.42	2.75
CNN_V	67.25	6.57
FCNN	92.50	3.05
MCF-CNN	97.75	1.92
ECNN	98.17	1.74
